# Postoperative Chemoradiotherapy versus Radiotherapy Alone in Major Salivary Gland Cancers: A Stratified Study Based on the External Validation of the Distant Metastasis Risk Score Model

**DOI:** 10.3390/cancers14225583

**Published:** 2022-11-14

**Authors:** Wenbin Yan, Lili Huang, Jianyun Jiang, Chunying Shen, Xiaomin Ou, Chaosu Hu

**Affiliations:** 1 Department of Radiation Oncology, Fudan University Shanghai Cancer Center, Shanghai 200032, China; 2 Department of Oncology, Shanghai Medical College, Shanghai 200032, China; 3 Shanghai Clinical Research Center for Radiation Oncology, Shanghai 200032, China; 4 Shanghai Key Laboratory of Radiation Oncology, Shanghai 200032, China

**Keywords:** salivary gland malignancy, chemoradiotherapy, external validation

## Abstract

**Simple Summary:**

Postoperative radiotherapy (PORT) was strongly recommended for major salivary gland malignancies (SGM) with adverse features. However, distant metastasis (DM) remained the major failure pattern of the patients, and the role of adjuvant chemotherapy was inconclusive. This study aimed to compare the survival outcome between the patients following adjuvant CRT or RT alone based on a DM risk score model. No significant difference was revealed between the CRT and RT group in the entire cohort when matching with the IPTW. After DM risk stratification, we found inferior survival with the administration of adjuvant CRT in the low-risk subset but no significant difference in the high-risk group. Our finding provided evidence that additional chemotherapy to PORT is not well recommended in clinical practice for major SGM patients.

**Abstract:**

Background: The role of additional chemoradiotherapy (CRT) for distant metastasis (DM) on the resected malignancy of the major salivary gland (SGM) remained unknown. We conducted this study to externally validate a recently reported DM risk score model and compare the survival outcome between adjuvant CRT and RT alone. Materials: We retrospectively reviewed the patients with SGM following postoperative radiotherapy (PORT). The cumulative incidence of DM was assessed using a competing risk method. Multivariate analysis was performed with Cox proportional-hazards regression to identify significant predictors for DM. Patients were classified as high- and low-risk subgroups with the cutoff value of the DM risk score model. The inverse probability of treatment weighting (IPTW) was conducted to minimize the bias of the groups. Results: A total of 586 eligible patients were analyzed and 67 cases underwent adjuvant CRT. The 5-year incidence of DM was 19.5% (95% CI 16.0–23.0%). The model reasonably discriminated the DM risk between the high- and low-risk subgroup in our cohort, and the c-index was 0.75. No survival benefit was observed for the CRT group compared with RT alone in the entire cohort after IPTW (*p* = 0.095). After subgroup analysis, increased mortality was identified with the administration of CRT in the low-risk subset (*p* = 0.002) while no significant difference in OS was illustrated in the high-risk subgroup (*p* = 0.98). Conclusions: This external validation provides further exploration of the DM risk score model in major SGM. Our results demonstrated no support for the utility of additional chemotherapy to PORT in the major SGM, especially in the low-risk subgroup of patients with DM.

## 1. Introduction

Adequate surgical resection is the definitive therapeutic approach for patients with major salivary gland malignancies (SGM). Postoperative radiotherapy (PORT) was associated with improvement in locoregional control and overall survival (OS) [1,2] for patients with adverse features such as specific sub-types [3], advanced stages [4], and high-risk pathological factors [5]. Thus, distant metastasis (DM) remained the most failure pattern that contributed to cancer-related mortality in major SGM after curative treatment [6,7]. The reported 5-year cumulative incidence of DM was 20–30% [4,8], which was the rationale for the utility of adjuvant chemotherapy. 

Postoperative concurrent chemoradiotherapy (CCRT) offered an improvement in local-control rate, progression-free survival (PFS), and OS for high-risk head and neck squamous cancer (HNSC) [9,10]. Nonetheless, the SGM comprised over 20 different histological types according to the new World Health Organization classification [11] and was rarely included in the studies of HNSC. Retrospective studies based on public databases have demonstrated no significant survival advantages for adjuvant chemoradiotherapy (CRT) of resected SGM [2,12]. However, increased overall survival (OS) was revealed for specific pathologies such as ACC and squamous cell carcinoma [13,14]. The pathological diversity and low incidence of SGM contributed to the difficulty in raising consensus on the role of CRT [15]. Identifying the subset of patients at high risk of DM [16] is essential for investigating the value of additional chemotherapy in the SGM following PORT.

The risk of DM after surgery varied owing to the heterogeneity of histological subtypes in SGM [7]. Administration of CRT in the subset of patients at high risk of DM could potentially improve survival, whereas overtreatment of chemotherapy may result in additional treatment-related toxicity and reduce survival benefits for the patients at low risk. Developing an effective and validated DM risk model was challenging due to the relative rarity of SGM. A recently reported DM risk score model [17] established by the Princess Margaret Cancer Center in an international multicenter study demonstrated favorable discrimination of the DM risk among major SGM. 

In this study, we sought to externally validate the DM risk score model based on the resected major SGM treated with PORT at a Chinese academic cancer center. Moreover, we aimed to compare the survival outcome of adjuvant CRT versus RT alone in the high- and low-risk subgroup of patients stratified by the model.

## 2. Materials and Methods

### 2.1. Participants of the Study

This study was approved by the Shanghai Cancer Center institutional review board (SCCIRB). Clinical information of patients with pathologically confirmed SGM treated at Fudan University Shanghai Cancer Center (FUSCC) between 2008 and 2020 was collected. The criteria of eligible patients included high-risk pathology, grades 2 to 3 disease, high-risk features (T3–4, N+, positive PNI or surgical margin). Additionally, we only included the patients who received primary tumor resection followed by adjuvant radiotherapy. Specific exclusion criteria are as follows: (1) with distant metastasis at initial diagnosis; (2) no treatment of surgery or radiotherapy; (3) no completion of the radiotherapy; and (4) missing data on the follow-up.

### 2.2. Histology

The histology was reviewed routinely in our institution and those confirmed as primary malignancies of the salivary gland according to the WHO were all enrolled. Squamous cell carcinoma (SCC) was excluded as most cases derived from the metastasis of node or head and neck SCC of the skin. For the lymphoepithelial carcinoma (LEC), nasopharyngeal endoscopy or biopsy was conducted to exclude the primary nasopharyngeal tumor. 

### 2.3. Treatment and Definitions

Adjuvant radiotherapy was performed after the multidisciplinary discussion using intensity-modulated radiation therapy (IMRT) or 3D-CRT once daily five times per week. Radiotherapy was delivered in the cases with at least one adverse feature including: T3–4, positive nodal involvement, positive surgical margin, high-risk pathology, perineural invasion (PNI) and lymphovascular invasion (LVI). Clinical target volumes covered the operative bed and involved nodal levels. Chemotherapy was delivered in sequential or concomitant settings in some highly selected patients as it was not recommended for conventional practice in major SGM. Potential chemotherapy criteria included late-stage and high-risk pathological features (positive surgical margin, late-stage nodal status). The platinum-based regimen was commonly used in the adjuvant CT while cisplatin alone as the concomitant schedule and sequential regimens including docetaxel and cisplatin (TP), cisplatin and 5-fluorouracil (PF), or docetaxel, cisplatin and 5-fluorouracil (TPF).

As an external validation of the DM risk score model, we classified the histological subtypes into two risk groups based on the previous study [17]. The pathological reports confirmed pathological characteristics involving grade of malignancy, perineural invasion (PNI), lymphovascular invasion (LVI), and surgical margin. 

### 2.4. Follow-Up

The diagnosis of DM was detected by biopsy or radiology including CT, MRI, or ECT. The time to DM was calculated from the end of radiotherapy to the date of distant metastasis on the follow-up imaging. The overall survival (OS) was defined as the time from the date of initial diagnosis to the death from any cause, or the last contact time. 

### 2.5. Statistical Analysis

The clinicopathological characteristics were summarized with descriptive statistics. Survival probability was estimated using the Kaplan–Meier method. The comparisons between variables were performed using the *t*-test or chi-square test. The association between variables and cumulative incidence of DM was identified with univariable analysis using Fine-Gray competing risk regression, and multivariable analysis was conducted with a Cox proportional hazards model. The cutoff value for the risk score of the high- and low-risk group was the same as the DM risk score model, and a concordance index (c-index) using Cox proportional-hazards regression was conducted to estimate the model. The inverse probability of treatment weighting (IPTW) [18] was used to minimize the bias of the clinical features between the pairwise groups. The statistical analyses were performed by IBM SPSS Statistic (version 26.0, IBM Corp, Armonk, NY, USA) and R software (version 4.0.5, Vienna, Austria). A two-tailed *p*-value of <0.05 was considered statistically significant.

## 3. Results

### 3.1. The Clinicopathological Characteristics

A total of 586 patients with major SGM treated from 2008 to 2020 at Fudan University Shanghai Cancer Center (FUSCC) were obtained. The median age for the patients at diagnosis was 49 years. The most common histology in our cohort was lymphoepithelial carcinoma (LEC) (*n* = 147), followed by adenoid cystic carcinoma (ACC) (*n* = 125), mucoepidermoid carcinoma (*n* = 81), and others (*n* = 257). All patients underwent surgical resection of the primary tumor, and neck dissection was conducted in 44.3% (260/586) of the patients. The PNI and LVI were found in 24.8% (*n* = 148) and 6.8% (*n* = 38) of the patients. Surgical margin involvement was observed in 29 cases (4.9%). More details about the clinical features of the enrolled patients are listed in Table 1.

### 3.2. Variables Associated with DM in the Major SGM Following PORT

In the univariable analysis, we found that age, N stage, histology, PNI, adjuvant CRT, and LVI were associated with DM in the major SGM. Furthermore, the multivariate analysis identified that nodal involvement (*p* < 0.001), high-risk histology (*p* = 0.015), PNI (*p* = 0.007), and LVI (*p* = 0.004) were independent predictors for DM (Table 2). The risk coefficients in our study were pN+ (1.2), high-risk histology (0.9), PNI (0.5), and LVI (0.8).

### 3.3. Comparison of Survival Outcome between the Adjuvant RT and CRT-Group

The median follow-up time of the entire cohort was 63.5 months (range 4.5–165), and 109 (18.6%) patients presented with DM during follow-up. The 5-year cumulative incidence of DM was 19.5% (95% CI 16.0–23.0%). The estimated 5- and10-year OS in the entire cohort was 85.4% and 70.7%, respectively. 

**Figure 1:**
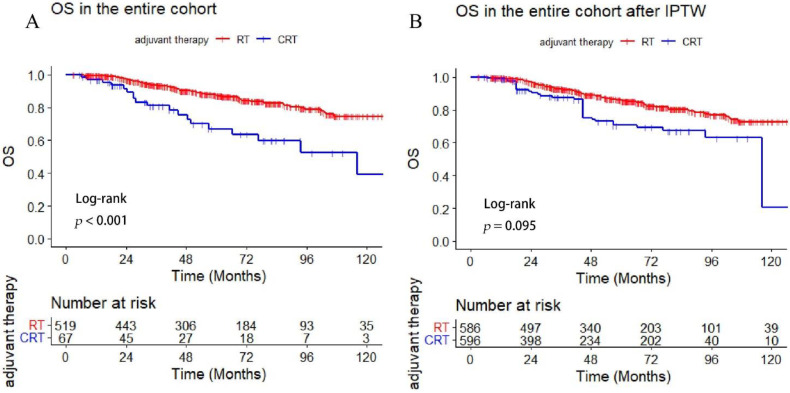
Comparison of survival between the adjuvant CRT and RT group (**A**) OS in the entire cohort, (**B**) OS comparison between treatment groups after IPTW.

Adjuvant chemotherapy was administrated in 11.4% (*n* = 67) of the patients. In addition, significantly poor survival was revealed in the CRT group compared with RT alone group (5-year OS: 67.0 versus 87.4%, *p* < 0.001). However, patients in the CRT group presented with a higher proportion of late-stage N and high-risk histology (*p* < 0.001, *p* < 0.001). More details about the baseline of the RT and CRT-group are summarized in Table 3. Moreover, we used the IPTW matching with the clinical factors (sex, age, T stage, N stage, pathology, and PNI) and found no statistical difference in OS between the two treatment groups (*p* = 0.095) (Figure 1).

### 3.4. External Validation of the Prediction-Score Model

**Figure 2:**
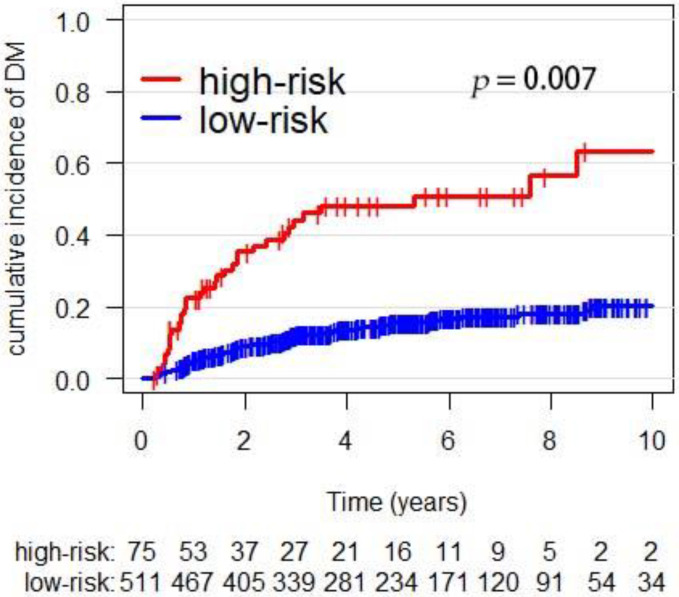
The incidence of DM in the DM risk subgroups.

To validate the DM risk score model, we used the risk coefficients: high-risk histology (1.2), pN+(0.7), pT3–4 stage (0.7), LVI (0.8), and margin (0.6) [17]. The high-risk group was defined as a sum of the score more than 2.0. Thus, the 5-year cumulative incidence of DM in the high-, and low-risk group was 48.0% and 15.6% (*p* = 0.007), respectively (Figure 2). The C-index for the model in the validation cohort for predicting DM was 0.75.

### 3.5. Association of Adjuvant CRT with Survival in High- and Low-Risk Subgroups

**Figure 3:**
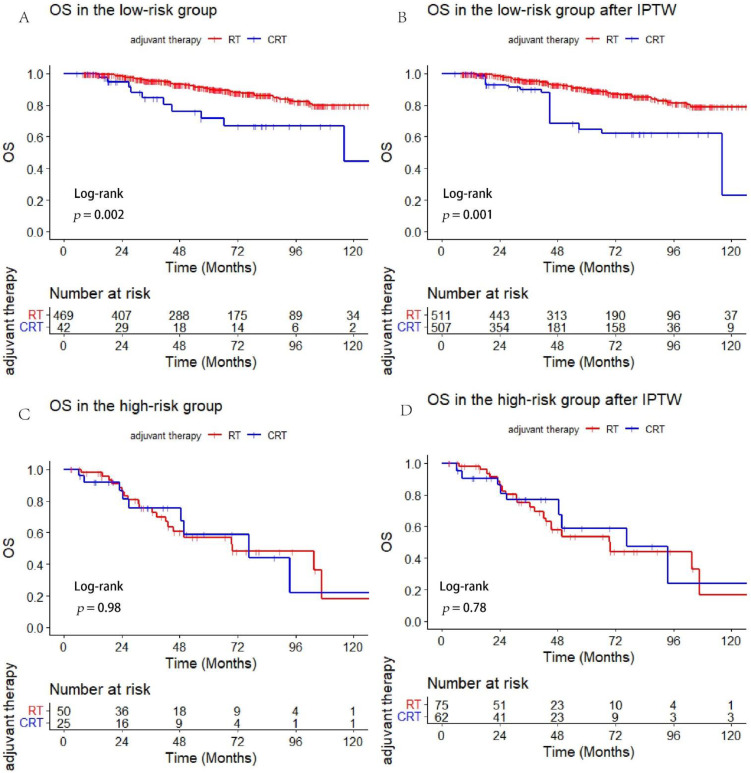
Comparison of OS between adjuvant CRT versus RT alone in the high- and low-risk group ((**A**): OS in the low-risk group, (**B**): OS in the low-risk after IPTW, (**C**): OS in the high-risk, (**D**): OS in the high-risk after IPTW).

Patients were divided into high- and low-risk subgroups based on the DM risk score model. Adjuvant CRT appeared to offer no survival benefits in high-risk patients (*p* = 0.98). However, increased mortality was associated with CRT for OS (*p* = 0.002) in the low-risk subgroup (Figure 3A,C). Moreover, we matched the two treatment groups with IPTW and found no survival difference in the high-risk group (*p* = 0.78) but decreasing OS for the patients following CRT in the low-risk group (*p* = 0.001) (Figure 3B,D). 

In the subgroup of patients with LEC, no significant difference in OS was found between the CRT and RT groups (*p* = 0.86). In addition, the similar result was obtained after IPTW matching with the T stage, N stage, sex, age, PNI, and LVI. Additionally, adjuvant CRT was associated with poor OS in the SDC subtype (*p* < 0.001) (Supplementary Figure S1). 

## 4. Discussion

We retrieved the clinical data of major SGM patients from the Chinese population to externally validate the DM risk score model and compare the survival outcome between the patients following adjuvant CRT or RT alone. Our results demonstrated that the DM risk score model could be applied to the Chinese population with major SGM. Additionally, we found no survival benefits for CRT in the cases at high risk of DM, whereas CRT was associated with increased mortality in the low-risk group. This finding may provide the evidence of no support for the utilization of adjuvant CRT in resected major SGM but a negative prognostic impact in the low-risk cohort.

The discovery cohort of the DM risk score model enrolled the participants with major SGM after curative surgery, and the risk score was calculated by incorporating the clinical factors such as histology, tumor stage, LVI, and surgical margin [17]. The c-index for the risk groups in the discovery and validation cohort were 0.75 and 0.79, respectively. Additionally, about 73% of the entire cohort underwent postoperative radiotherapy (PORT) and 5-year cumulative incidence of DM was 20%, which is consistent with the result in our study (5-year DM: 19.5%). Furthermore, the external validation showed favorable calibration with the c-index of 0.75 for predicting DM following PORT. Several other predictors associated with DM were not included in the constructed model. PNI was an independent factor correlated with worse OS and DFS [6,19] in the major SGM following PORT. Another study [20] has suggested that PNI was associated with a high risk of DM, as shown by the multivariate analysis in our study (*p* = 0.007). However, no statistical significance was identified from the multivariate analysis of the DM risk score model. The possible reasons included the diversity of histological subtypes in major SGM, variability in the details of clinical or pathological invasion [21], and resection situation. Moreover, studies with a small sample size have found that the tumor site of the sublingual gland was correlated with poorer DMFS than the site of the parotid [22,23]. Therefore, more effective predicting models for DM with sufficient samples still need to be explored. 

Though lacking prospective evidence for adjuvant radiotherapy, several retrospective studies [3,24,25] involving large cohorts of patients have illustrated the survival improvement for adjuvant RT after surgery. Nonetheless, the optimal role and criteria for adjuvant chemotherapy with RT in the resected major SGM remained unclear. Thus, the adjuvant CRT was rarely administrated in clinical practice, and only 11.8% of the patients received adjuvant CRT in this cohort. A similar proportion (6–10.8%) was observed in several other studies with large cohorts [2,17], and no survival benefit was found with the CRT in the major SGM. Concurrent RT with cisplatin alone significantly improved OS and PFS for high-risk squamous-cell carcinoma of head and neck [9,10]. In addition, cisplatin became the main regimen for CCRT used in major SGM. However, no survival advantages were observed with the additional CCRT to adjuvant therapy compared to RT alone [12,26]. The pathological heterogeneity in SGM, variability in sensitivity to chemotherapy, and tolerance of patients were all to be evaluated when considering the delivery of adjuvant chemotherapy. 

Identification of the specific subsets with high-risk factors is critical when considering the addition of chemotherapy with RT. Given the high proportion of advanced N stage and high-risk histology in the CRT group, significantly poor OS was revealed when compared with the RT alone group. Nonetheless, no benefit of CRT was shown in the entire major SGM after IPTW matching. However, adding CRT to operation was associated with increased survival for late-stage salivary squamous-cell carcinoma [14]. In addition, adjuvant CRT could improve local-regional control for ACC compared with RT alone [13]. Salivary duct carcinoma (SDC) is a highly aggressive subtype of salivary tumors, but no benefit of OS and DMFS was identified with the addition of CRT in patients with SDC [27], which was consistent with our results for the SDC. Besides the specific pathological types, several clinical factors should be integrated to discriminate the potentially high-risk individuals. Two studies [28,29] have demonstrated significant survival improvement of CRT in the subgroup with stage III or IV, PNI, positive surgical margin, facial nerve involvement, high-grade histology, and extra-glandular. However, the small sample sizes of these studies make the findings inconclusive. Furthermore, a study with large samples from Surveillance, Epidemiology, and End Results (SEER) database has revealed worse OS for CRT versus RT in elderly SGM patients after surgery [30]. The inclusion criteria of CRT in our study included cisplatin alone in the concurrent set and platinum-based multiagent in the sequential set, and no significance in OS was illustrated after IPTW analysis. Moreover, we defined the high-risk subset of patients based on the external validation for an effective DM risk score model. Equivalent OS was identified in the high-risk subgroup of patients, whereas the utility of CRT was associated with a higher risk of mortality in patients at low risk of DM. Our findings supported the limited utilization of additional chemotherapy for low-risk patients, and more robust evidence for CRT await high-quality prospective studies.

To our knowledge, this is the first study to externally validate the DM risk score model of the Princess Margaret Cancer Center based on the Chinese population. Nonetheless, we acknowledged that several limitations should be considered with the results of this study. First, inherent selection bias may exist owing to the retrospective design of the work. Second, the toxicity of adding chemotherapy was not documented in the study. Other limitation includes the potential bias of treatment for major SGM on surgery and RT in a single institution.

## 5. Conclusions

We presented this study to validate the efficacy of the DM risk score model in major SGM following PORT among the Chinese population. Furthermore, inferior survival was demonstrated in the low-risk subset of patients while no significant difference was revealed in the high-risk group with the utility of CRT for resected SGM. The administration of additional chemotherapy to PORT is not well-recommended in clinical practice for major SGM patients.

## Figures and Tables

**Table 1:** The clinicopathological features of the patients in the external validation.

	FUSCC Cohort (*n* = 586, %)
Sex	
Male	350 (60.3)
Female	236 (39.4)
Age (median, range)	49 (18–83)
Tumor Site	
Parotid	421 (72.5)
Submandibular/sublingual	165 (27.5)
p T stage	
T1–2	470 (80.5)
T3–4	116 (19.5)
p N stage	
N0	406 (68.5)
N+	180 (31.5)
Stage	
I	119 (20.0)
II	219 (37.0)
III	98 (16.9)
IVa	138 (24.1)
IVb	12 (2.0)
PNI	
Negative	438 (75.2)
Positive	148 (24.8)
LVI	
Negative	548 (93.2)
Positive	38 (6.8)
Margin	
Negative	557 (95.1)
Positive	29 (4.9)
Pathology	
low-risk	143 (23.6)
high-risk	443 (76.4)
PTV (Gy)	60 (45–70.4)
Adjuvant therapy	
RT alone	519 (88.2)
CRT	67 (11.8)
Histology	
Lymphepithelioma carcinoma (LEC) ^a^	147 (25.1)
Adenoid cystic carcinoma	125 (21.3)
Mucoepidermoid carcinoma	81 (13.8)
Salivary duct carcinoma	79 (13.5)
Acinic cell carcinoma	37 (6.3)
Carcinoma ex-pleomorphic adenoma ^a^	25 (4.3)
Adenocarcinoma	18 (3.1)
Others ^a^	74 (12.6)

Abbreviations: PNI: perineural invasion, LVI: lymphovascular invasion, CRT: chemoradiotherapy. a: the complete break-down of histology was listed in the Supplementary Table S1.

**Table 2:** Univariable and multivariate analysis for DM with a competing-risk method (*n* = 586).

	Univariable Analysis		Multivariate Analysis	
	HR (95% CI)	*p*-Value	HR (95% CI)	*p*-Value
Age	1.02 (1.01–1.04)	<0.001	1.01 (1.00–1.03)	0.05
Gender		0.109		
Male	Ref.			
Female	0.72 (0.48–1.07)			
T stage		0.087		
T1–2	Ref.			
T3–4	1.44 (0.95–2.21)			
N stage		<0.001		<0.001
N0	Ref.		Ref.	
N+	4.29 (2.92–6.30)		3.36 (2.25–5.03)	
Histology		<0.001		0.015
Low-risk	Ref.		Ref.	
High-risk	3.88 (1.96–7.65)		2.51 (1.19–5.30)	
PNI		<0.001		0.007
Negative	Ref.		Ref.	
Positive	2.42 (1.65–3.54)		1.77 (1.168–2.67)	
LVI		<0.001		0.004
Negative	Ref.		Ref.	
Positive	3.92 (2.30–6.68)		2.35 (1.31–4.23)	
Surgical margin		0.81		
Negative	Ref.			
Positive	0.88 (0.32–2.41)			
CRT		0.001		0.7
No	Ref.		Ref.	
Yes	2.42 (1.46–4.03)		1.11 (0.65–1.89)	

Abbreviations: PNI: perineural invasion, LVI: lymphovascular invasion, CRT: chemoradiotherapy.

**Table 3:** The baseline characteristics in the RT and CRT groups.

	RT (*n* = 519, %)	CRT (*n* = 67, %)	*p*-Value
Sex			0.898
Male	309 (59.5)	41 (61.2)	
Female	210 (40.5)	26 (38.8)	
Age	47.8 ± 14.7	49.0 ± 14.1	0.537
T stage			0.466
T1–2	419 (80.9)	51 (76.1)	
T3–4	100 (19.3)	16 (23.9)	
N stage			<0.001
N0	394 (75.1)	12 (17.9)	
N+	125 (24.9)	55 (82.1)	
PNI			0.637
Negative	390 (75.1)	48 (71.6)	
Positive	129 (24.9)	19 (28.4)	
LVI			0.064
Negative	489 (94.2)	59 (88.1)	
Positive	30 (5.8)	8 (11.9)	
Margin			0.128
Negative	496 (95.6)	61 (91.0)	
Positive	23 (4.4)	6 (9.0)	
Pathology *			<0.001
low-risk	139 (26.8)	4 (5.9)	
high-risk	380 (73.2)	63 (94.1)	

Abbreviations: PNI: perineural invasion, LVI: lymphovascular invasion, CRT: chemoradiotherapy. *: the complete break-down of the histological subtypes was listed in Supplementary Table S2.

## Data Availability

The datasets used and/or analyzed during the current study available from the corresponding author on reasonable request.

## Supplementary Materials

The following supporting information can be downloaded at: https://www.mdpi.com/article/10.3390/cancers14225583/s1, Supplementary Figure S1: Overall survival comparison between RT and CRT group in the LEC and SDC subtype; Supplementary Table S1: Histology of the salivary gland carcinoma in the entire cohort, Supplementary Table S2: Histological subtypes in the RT and CRT group.
