# Supplementary material for: Postoperative Chemoradiotherapy versus Radiotherapy Alone in Major Salivary Gland Cancers: A Stratified Study Based on the External Validation of the Distant Metastasis Risk Score Model
Source: Cancers (Basel). 2022 Nov 14;14(22):5583. doi: 10.3390/cancers14225583 (PMC9688786; doi:10.3390/cancers14225583)

Supplementary material Table S1 Histology of the salivary gland carcinoma in the entire cohort

	**FUSCC cohort (n=586, %)**
Histology	
Lymphoepithelial carcinoma (LEC)	147 (25.1)
Adenoid cystic carcinoma (ACC)	125 (21.3)
Mucoepidermoid carcinoma (MEC)	81 (13.8)
Salivary duct carcinoma (SDC)	79 (13.5)
Acinic cell carcinoma	37 (6.3)
Carcinoma ex-pleomorphic adenoma	25 (4.3)
Adenocarcinoma	18 (3.1)
Secretory carcinoma	13 (2.2)
Carcinoma NOS	26 (4.4)
Myoepithelial carcinoma	18 (3.1)
Small cell carcinoma	8 (1.4)
Basel cell adenocarcinoma	6 (1.0)
Carcinosarcoma	3 (0.5)

In the cases with LEC, Epstein-Barr virus was positive in 71 patients, negative in 30 cases and unknown in 46 cases. The carcinoma ex-pleomorphic adenoma was consisted of 13 patients with high-grade SDC, and 12 myoepithelial carcinoma cases.

Supplementary material Table S2 Histological subtypes in the RT and CRT group

	RT (n=519, %)	CRT (n=67, %)	P-value
Pathological subtype			<0.001
LEC	120 (23.1)	27 (40.3)	
ACC	122 (23.5)	3 (4.5)	
MEC	80 (15.4)	1 (1.5)	
SDC	66 (12.7)	13 (19.4)	
Acinic cell carcinoma	37 (7.1)	0 (0)	
ex-pleomorphic adenoma	22 (4.2)	3 (4.5)	
Adenocarcinoma	13 (2.5)	5 (7.5)	
Secretory carcinoma	13 (2.5)	0 (0)	
Carcinoma NOS	20 (23.1)	6 (9.0)	
Myoepithelial carcinoma	15 (2.9)	3 (4.5)	
Other	11 (2.1)	6 (9.0)	

Supplementary Figure S1 Overall survival comparison between RT and CRT group in the LEC and SDC subtype

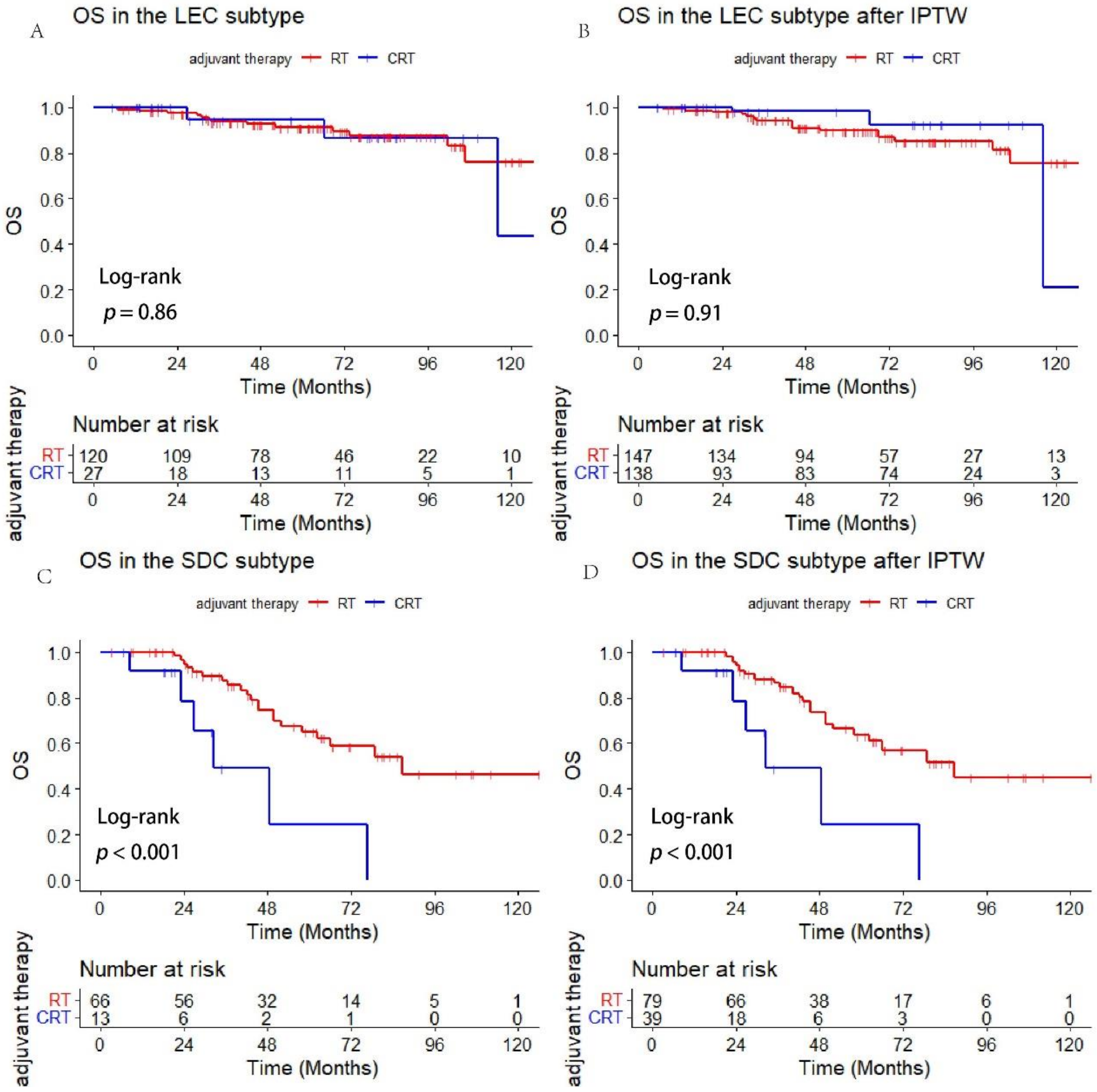

Supplement: Supplementary file 1 [file cancers-14-05583-s001.zip › cancers-1848025-supplementary.pdf]
